# Sexual Harassment and Mental Health of Female Healthcare Workers in a Tertiary Care Hospital in Riyadh, Saudi Arabia

**DOI:** 10.7759/cureus.30860

**Published:** 2022-10-29

**Authors:** Hind Abdullah Aloraier, Rawan Mousa Altamimi, Elham Ahmed Allami, Razan Abdullah Alqahtani, Taif Shabib Almutairi, AlJohara M AlQuaiz, Ambreen Kazi, Eman Alhalal

**Affiliations:** 1 Princess Nora Bint Abdullah Chair for Women’s Health Research, King Saud University Medical City, Riyadh, SAU; 2 Family and Community Medicine, King Saud University Medical City, Riyadh, SAU; 3 Community and Mental Health, College of Nursing, King Saud University Medical City, Riyadh, SAU

**Keywords:** healthcare workers, mental wellbeing, low self-esteem, saudi females, sexual harassment

## Abstract

Background

In this study, we aimed to estimate the prevalence and identify the correlates of sexual harassment among female healthcare workers in a tertiary care hospital in Riyadh, Saudi Arabia.

Methodology

A cross-sectional study was conducted among 432 female healthcare workers in both Arabic and English languages. The questionnaire consisted of five sections which included the sociodemographic characteristics, a sexual harassment survey, the Kessler distress scale, social support, and the Rosenberg self-esteem scale. Unadjusted odds ratios with 95% confidence intervals were calculated to identify the significant factors associated with harassment.

Results

Sexual harassment was reported by 15.5% of the participants, with verbal harassment being the most commonly reported (66%), followed by physical harassment (34%). Around three-fourths of harassment acts took place during the daytime, and 18% of the participants preferred to keep quiet about it. Moderate distress [2.38 (1.17, 4.84)] and severe distress [2.31 (1.09, 4.90)], feeling hopeless [2.86 (1.47, 5.57)] and feeling depressed [3.70 (1.62, 8.48)] were significantly associated with sexual harassment. Low self-esteem items, such as “I don’t have good qualities” [4.78 (2.0, 11.43)], “don’t have much to be proud of” [2.10 (1.22, 3.63)], “wish that I have more respect for myself” [2.30 (1.36, 3.90)], and “inclined to feel that I am a failure” [2.27 (1.24, 4.16)] were significantly associated with sexual harassment.

Conclusions

It is important for all employees to know about their rights and report all types of harassment acts. Counseling sexually harassed victims should focus on improving the self-esteem and mental distress of these women. Prevention of harassment against female healthcare workers can improve their mental health as well as their quality of work.

## Introduction

Workplace sexual harassment is unacceptable behavior of unwanted sexual action which the attacker uses against someone [1], which may be verbal or physical. It is a prevalent issue across countries with women being the main victims [2]. For example, in Nepal, 42% of female healthcare workers encountered sexual harassment at the workplace [3], while in the United States, approximately 30% of female residents experienced workplace sexual harassment [4]. A systematic review revealed that 43.15% of female nurses are a target of workplace sexual harassment [5]. These differences in prevalence, though not fully explained, might be due to cultural variations and sensitivity in reporting the issue.

According to some theories, workplace sexual harassment originates from an individual’s sex and gender role, where the harasser is trying to promote their own sex-based social status, enhance hierarchical structure [6], or reinforce power and control [7]. Another perspective assumes that organizational context and tolerance can perpetuate sexual harassment [8]. Thus, some studies examined the factors that can make a person more susceptible to sexual harassment in different countries [9,10]. Being female [11], young age, not having children [12], being pretty, and having a friendly character [13] are factors that might increase the risk of workplace sexual harassment. In addition, working the night shift increases the odds of experiencing sexual harassment [14]. Although a majority of studies have examined the risk factors of workplace violence in the context of health care and included sexual harassment as one of its types [15-17], most existing studies are from North America [4,18] and Europe [19,20], which may not be applicable to other countries (such as Saudi Arabia) because sociocultural factors influence the prevalence and perception of the issue [21].

Sexual harassment is a significant health hazard in work settings. Workplace harassment is associated with poorer well-being in comparison with public violence [22]. It is associated with an increased risk of psychological health issues, such as anxiety, depression, posttraumatic stress disorder, poor psychological well-being, sadness, disgust, and sleep problems [23]. It also increases the likelihood of burnout, poor self-esteem, and self-confidence [24]. Harassment also has a huge influence on work functioning aspects [25], such as poor job satisfaction [24], absenteeism and psychological distress [26], intention to leave [27], and decreased productivity [28].

According to a recent study conducted in Saudi Arabia, the prevalence of sexual harassment was 19.3% and was experienced more often by female residents [29]. Furthermore, Saudi Arabia has transformed into a moderate society, and the vision of 2030 aims to have more women in the workforce. However, there is not sufficient literature on the prevalence and associated factors of sexual harassment in a healthcare setting. The objectives of this study were to determine the prevalence of harassment, identify the most common type, and measure the factors associated with sexual harassment in female healthcare workers in a tertiary care hospital in Riyadh, Saudi Arabia. Hence, this study contributes to preventing the increasing incidence of sexual harassment, drawing the stakeholders’ attention to raise awareness, encouraging women to speak up and make a formal complaint against the harasser, and overcoming external inhibitors such as the culture and society.

## Materials and methods

This cross-sectional study was conducted from September 2019 to April 2020 in a public tertiary care hospital in Riyadh city. Keeping the prevalence of sexual harassment among female healthcare workers around 40% [3], with a 95% confidence interval level and 5% precision, the calculated sample size was 375. By adding 20% to cover the non-response rate, the total sample size required was 450. The sampling frame comprised all full-time female healthcare workers, including doctors, nurses, technicians, laboratory staff, health educators, pharmacists, and dieticians, aged 20 to 59 years, working as regular employees. The sample was selected through a convenient sampling technique using both face-to-face interviews and online (sending out emails). The research proposal (19-4455) was approved by the Institutional Review Board. All participants read and signed the online consent form before answering the questionnaire.

Data collection and measurements

For data collection, 450 questionnaires were distributed manually and online among female healthcare workers working in different medicine and allied wards, surgical wards, dental clinics, family medicine clinics, pediatric clinics, laboratories, radiological departments, nutritional departments, inpatient and outpatient pharmacies, and lounge rooms within the hospital. The response rate was 96% as 432 forms were completely filled, returned, and included in the analysis.

All scales included in the study were originally developed in the English language and later translated into the Arabic language. The questionnaire consisted of five sections. The first section included information about the sociodemographic characteristics of the participants, including age, nationality, marital status, type of health worker, level of training, and years of experience. The second section included questions on harassment in the workplace [30]. The questions on sexual harassment utilized in a previous survey study [30] were modified according to the objectives of this study. The questions included a description of the sexual harassment experience; the type, time, and frequency; characteristics of the harasser; response; and the action taken. The questions were pretested on a group of women for clarity and to avoid duplication.

The third section consisted of the Kessler Psychological Distress Scale, commonly known as K-10 [31,32]. K-10 is a global measure questionnaire for measuring mental distress. It consists of 10 items about anxiety and depressive symptoms over the past four weeks. The responses are graded on a Likert scale, ranging from none to all the time. The scores range from 10-50, with a score <20 indicating that the person is well, a score between 20 and 24 indicating mild distress, a score between 25 and 30 indicating moderate distress, and a score >30 indicating severe distress. The Cronbach alpha value for K-10 was 0.88.

The fourth section consisted of the Social Support Scale [33]. It consists of four functional social support questions, two questions are about emotional support which are if you have someone to listen to you, and if you have someone to discuss problems with. The other two questions are about tangible support which are if you have someone to take you to the doctor, and if you have someone to help you with daily housework. The Cronbach alpha for the scale was 0.78.

The fifth section included the Rosenberg Scale for Self-Esteem (RSES) [34]. The RSES is a global scale that measures self-confidence by measuring positive and negative emotions using 10 questions, with each question having four options, following Likert responses which range from strongly agree to strongly disagree. The responses are summed to obtain a total score ranging from 10 to 40, with high scores indicating high self-esteem. The Cronbach’s alpha was 0.77.

Data analysis

The data were analyzed using SPSS version 25 (IBM Corp., Armonk, NY, USA) [35]. Descriptive and analytical statistics were done to calculate the frequencies and percentages for categorical variables. The Likert scale responses for the self-esteem scale were converted to dichotomous variables by adding the agree and strongly agree as one response versus disagree and strongly disagree as one response. Similarly, social support responses were categorized as social support available all the time or most of the time as the reference category and coded as 0 versus available sometimes or no social support coded as 1. Pearson chi-square test was used to find the association between mental distress, self-esteem, social support, demographic factors, and sexual harassment in the workplace. Unadjusted odds ratios with 95% confidence intervals (CIs) were calculated. P-values of <0.05 were used to report the statistical significance and the precision of the results.

## Results

In total, 432 data forms were included in the analysis. Table 1 shows the association between sociodemographic variables and sexual harassment. Sexual harassment was reported by 15.5% (n = 67) of women. The majority of female healthcare workers were Saudi nationals, married, and aged ≥30 years. By profession, most were nurses (61%), followed by physicians/dentists (22%), technical support staff (17%), and working in the hospital for >5 years. There was no statistically significant difference between the harassed and not-harassed groups regarding sociodemographic characteristics.

**Table 1:** Association between sociodemographic characteristics and sexual harassment in female healthcare workers in a tertiary care hospital in Riyadh, Saudi Arabia (N = 432).

Characteristics	Harassed, n = 67 (15.5%)	Not harassed, n = 365 (84.5%)	Unadjusted odds ratio (95% CI)	P-value
Age (in years)				
30 and above	36 (53.7)	220 (60.3)	1.0	0.32
20–29	31 (46.3)	145 (39.7)	1.31 (0.77, 2.21)	
Nationality				
Saudi	30 (44.8)	150 (41.1)	1.0	0.57
Non-Saudi	37 (55.2)	215 (58.9)	0.77 (0.46, 1.29)	
Marital status				
Married	30 (44.8)	201 (55)	1.0	0.12
Single	37 (55.2)	164 (45)	1.50 (0.89, 2.54)	
Profession				
Physician/Dentists	18 (26.9)	78 (21.4)	1.0	0.52
Nurses	42 (62.7)	222 (60.8)	0.82 (0.45, 1.51)	
Technician/Support staff	7 (10.4)	65 (17.8)	0.47 (0.18, 1.19)	0.11
Years of working				
>5 years	38 (56.7)	218 (59.7)	1.0	
≥1–5 years	15 (22.4)	76 (20.8)	1.13 (0.59, 2.17)	0.71
<1 year	14 (20.9)	71 (19.5)	1.13 (0.58, 2.21)	0.72

Table 2 presents the details related to the acts of harassment. It was surprising to note that around 76% of the harassment acts took place during the daytime and while working in the wards or in the doctor’s room, with some even mentioning the nurse’s room. In more than 80% of cases, the harasser was a Saudi male person. Besides the “patient” as the harasser (reported by 33%), others included were doctors (28%), visitors (12%), and pharmacists (9%). Post-harassment action found that a majority (25%) confided in a colleague/family member, whereas a significant 18% preferred to keep quiet; however, around 19% reported the incident to their supervisor, and around 13% confronted the harasser. Appropriate action was taken by the supervisor against all reported complaints.

**Table 2:** Frequency percentages for workplace harassment reported by female healthcare workers in a tertiary care hospital in Riyadh, Saudi Arabia (N = 67).

Characteristics	N (%)
Type of harassment	
Verbal (sexual remarks)	44 (65.7)
Non-verbal (physical contact, unwanted touching, etc.)	23 (34.3)
Number of harassment acts	
Once	30 (44.8)
More than once	37 (55.2)
Time	
Night shift	16 (23.9)
Day shift	51 (76.1)
Gender of the harasser	
Male	57 (85.1)
Female	10 (14.9
Nationality of the harasser	
Saudi	56 (83.6)
Non-Saudi	11 (16.4)
Harassed by	
Doctors/Dentists	19 (28.5)
Pharmacist	6 (8.9)
Visitor	8 (11.9)
Patient	22 (32.8)
Others	12 (17.9)
Post-harassment action	
Kept quiet	12 (17.9)
Informed a friend/family member (outside the organization)	17 (25.4)
Informed a colleague	16 (23.9)
Reported it to the supervisor	13 (19.4)
Confronted him	9 (13.4)

Table 3 presents the association between mental distress (K-10 items) and harassment. There was a significant difference in the mean (±SD) distress scores between the harassed and un-harassed group [22.0 (±6.8) vs. 20.2 (±6.9), p = 0.05]. The total K-10 scores found that moderate distress [2.38 (1.17, 4.84)] and severe distress [2.31 (1.09, 4.90)] were significantly associated with harassment. Individual K-10 items found that feeling hopeless [2.86 (1.47, 5.57)] and feeling depressed [3.70 (1.62, 8.48)] were significantly associated with harassment.

**Table 3:** Association between mental distress and sexual harassment in female healthcare workers in a tertiary care hospital in Riyadh, Saudi Arabia (N = 432). No distress = score <20; mild distress =  score between 20 and 24; moderate distress = score between 25 and 30; severe distress = score >30.

K-10 items	Harassed, 67 (15.5%)	Not harassed, 365 (84.5%)	Unadjusted odds ratio (95% CI)	P-value
Distress category (total)				
No distress	31 (46.3)	221 (60.5)	1.0	
Mild distress	10 (14.9)	65 (17.8)	1.08 (0.51, 2.32)	0.81
Moderate distress	14 (20.9)	42 (11.5)	2.38 (1.17, 4.84)	0.02
High distress	12 (17.9)	37 (10.1)	2.31 (1.09, 4.90)	0.03
K-10 items (separate)				
Feel tired for no good reason				
No/Little	24 (35.6)	145 (39.7)	1.0	
Sometime	24 (35.6)	154 (42.2)	0.98 (0.52, 1.80)	0.94
All the time/Most of the time	19 (28.8)	66 (18.1)	1.80 (0.92, 3.54)	0.09
Feeling nervous				
No/Little	31 (47)	211 (57.8)	1.0	
Sometime	26 (38.1)	106 (29)	1.60 (0.90, 2.84)	0.11
All the time/Most of the time	10 (15.2)	48 (13.2)	1.41(0.65, 3.07)	0.39
Nervous that nothing can calm you				
No/Little	51 (75.8)	296 (81.5)	1.0	
Sometime	14 (21.2)	48 (13)	1.76 (0.90, 3.43)	0.10
All the time/Most of the time	2 (3)	21 (5.5)	.59 (.13, 2.60)	0.49
Feeling hopeless				
No/Little	46 (68.2)	299 (82.1)	1.0	
Sometime	16 (24.2)	38 (10.2)	2.86 (1.47, 5.57)	0.002
All the time/Most of the time	5 (7.6)	28 (7.7)	1.18 (0.43, 3.22)	0.74
Feeling restless and fidgety				
No/Little	41 (60.6)	261 (71.6)	1.0	
Sometime	21 (31.8)	79 (21.8)	1.73 (0.96, 3.10)	0.07
All the time/Most of the time	5 (7.6)	25 (6.6)	1.35 (0.49, 3.75)	0.56
So restless, you cannot stand it				
No/Little	54 (80.3)	309 (84.6)	1.0	
Sometime	9 (13.6)	38 (10.4)	0.73 (0.24, 2.26)	0.58
All the time/Most of the time	4 (6.1)	18 (5)	1.03 (0.28, 3.83)	0.96
Feeling depressed				
No/Little	34 (50.8)	278 (76.3)	1.0	
Sometime	23 (34.3)	65 (17.6)	2.93 (1.62, 5.31)	0.000
All the time/Most of the time	10 (14.9)	22 (6.1)	3.70 (1.62, 8.48)	0.002
Everything is an effort				
No/Little	21 (31.3)	138 (37.5)	1.0	
Sometime	13 (19.4)	87 (23.7)	0.98 (0.46, 2.05)	0.95
All the time/Most of the time	33 (49.3)	140 (38.8)	1.51 (0.83, 2.75)	0.17
Nothing can cheer you up				
No/Little	47 (69.7)	288 (78.9)	1.0	
Sometime	13 (19.7)	46 (12.6)	1.75 (0.87, 3.38)	0.11
All the time/Most of the time	7 (10.6)	31 (8.5)	1.39 (0.58, 3.35)	0.46
Feeling worthless				
No/Little	55 (81.8)	314 (86.1)	1.0	
Sometime	8 (12.1)	39 (10.6)	1.18 (0.52, 2.6)	0.69
All the time/Most of the time	4 (6.1)	12 (3.3)	1.92 (0.60, 6.17)	0.27

Table 4 shows a significant association between self-esteem items and harassment. Self-esteem responses strongly agree and agree were merged and coded as 0, whereas strongly disagree and agree were coded as 1. No significant difference was found in mean self-esteem scores between the two groups [19.9 (±5.3) vs. 20.8 (±4.2), p = 0.10]. However, items suggesting low self-esteem, such as “I don’t have good qualities” [4.78 (2.0, 11.43)], “don’t have much to be proud of” [2.10 (1.22, 3.63)], “wish that I have more respect for myself” [2.30 (1.36, 3.90)], and “inclined to feel that I am a failure” [2.27 (1.24, 4.16)] were significantly associated with harassment. Table 5 shows the association between social support and harassment. Availability of social support was more or less equally reported by both groups (88% and 84%, respectively). Similarly, the availability of tangible support was reported by 55% and 65% of female health workers. There was no significant association between social support and sexual harassment (p < 0.05).

**Table 4:** Association between self-esteem and sexual harassment in female healthcare workers in a tertiary care hospital in Riyadh, Saudi Arabia (N = 432).

Self-esteem	Harassed, 67 (15.5%)	Not harassed, 365 (84.5%)	Unadjusted odds ratio (95% CI)	P-value
I am satisfied with myself				
Agree	60 (89.6)	345 (94.6)	1.0	0.12
Disagree	7 (10.4)	20 (5.4)	2.01 (0.81, 4.95)	
I think I am no good at all				
Disagree	40 (59.7)	256 (70.1)	1.0	0.09
Agree	27 (40.3)	109 (29.9)	1.58 (0.92, 2.70)	
I feel that I have a number of good qualities				
Agree	57 (85.1)	351 (96.2)	1.0	0.00
Disagree	10 (14.9)	14 (3.8)	4.78 (2.0, 11.43)	
I am able to do things as well as most other people				
Agree	59 (88.2)	337 (92.3)	1.0	0.22
Disagree	8 (11.8)	28 (7.7)	1.62 (0.71, 3.73)	
I feel I do not have much to be proud of				
Disagree	39 (58.5)	273 (74.7)	1.0	0.007
Agree	28 (41.5)	92 (25.3)	2.10 (1.22, 3.63)	
I certainly feel useless at times				
Disagree	44 (65.7)	272 (74.5)	1.0	0.14
Agree	23 (34.3)	93 (25.5)	1.51 (0.87, 2.64)	
I feel that I am a person of worth, at least on an equal plane with others				
Agree	58 (86.6)	328 (89.9)	1.0	0.42
Disagree	9 (13.4)	37 (10.1)	1.38 (0.63, 3.0)	
I wish I could have more respect for myself				
Disagree	33 (49.3)	253 (69.3)	1.0	0.002
Agree	34 (50.7)	112 (30.7)	2.30 (1.36, 3.90)	
All in all, I am inclined to feel that I am a failure				
Disagree	48 (71.6)	311 (85.1)	1.0	0.007
Agree	19 (28.4)	54 (14.9)	2.27 (1.24, 4.16)	
I take a positive attitude toward myself				
Agree	59 (88.1)	340 (93.2)	1.0	0.15
Disagree	8 (11.9)	25 (6.8)	1.84 (0.79, 4.28)	

**Table 5:** Association between social support and sexual harassment in female healthcare workers in a tertiary care hospital in Riyadh, Saudi Arabia (N = 432).

Characteristics	Harassed, 67 (15.5%)	Not harassed, 365 (84.5%)	Unadjusted odds ratio (95% CI)	P-value
Emotional support				
Someone to listen/talk to				
Yes	63 (94)	327 (89.6)	1.0	0.25
No	5 (6)	38 (10.4)	0.54 (0.19, 1.57)	
Someone to discuss problems				
Yes	59 (88.1)	308 (84.4)	1.0	0.43
No	8 (11.9)	57 (15.6)	0.73 (0.33, 1.60)	
Tangible support				
Someone to take you to the doctor				
Yes	37 (55.2)	238 (65.2)	1.0	0.14
No	30 (44.8)	127 (34.8)	1.49 (0.88, 2.52)	
Someone to help with household chores				
Yes	45 (67.2)	246 (67.4)	1.0	0.98
No	22 (32.8)	119 (32.6)	1.0 (0.57, 1.73)	

## Discussion

This study was conducted to describe workplace sexual harassment among female healthcare workers in a public hospital in Saudi Arabia. The prevalence of sexual harassment in our study (15.5%) was less than that reported by a previous Saudi study (19.3%) [29]. It is interesting to note that though Korea has a different social and cultural environment, the prevalence in both countries is comparable (17.9% vs 15.5%) [36], thus emphasizing that workplace harassment is indeed an international public health issue.

We found that around 15.5% of female healthcare workers, mainly nurses, experience sexual harassment, with the verbal form being the most frequently reported type. This can be explained by the power relations and hierarchical environment in the healthcare system which considers consultants as superior and nurses as inferior, thus exposing them to experience harassment from physicians and patients [30,37]. This highlights the need of a working environment where a horizontal flow of power exists [38]. A study conducted in Saudi Arabia showed that most of the harassers were consultants which may be because they have authority over the medical team [29], which is different from other studies done in Egypt [39], Nepal [40], and Brazil [41], which showed that most of the harassers were the relatives and visitors of the patients. Some attendants accompanying the patients feel they have authority over nurses and health workers, thus they consider passing verbal comments and harassing them as their right.

Similar to previous studies, experiencing verbal harassment was more common in comparison to non-verbal (66% vs 34%) [5,36,39]. The global tendency toward verbal harassment suggests that verbal abuse is one of the most convenient and easiest ways to harass. Interestingly, in some cultures, victims of verbal harassment do not consider inappropriate comparisons or expressions of sexual appeal as a form of harassment [36]. Thus, harassers do not let the victim realize this is even an act of harassment. The difficulty of proving verbal harassment and the difficulty to respond to it by victims makes it most easy to practice. In some cases, extreme forms of non-verbal harassment have been observed. A study conducted in Tanta in a university hospital in Egypt found that the most common type of sexual harassment was staring in a suggestive manner [42]. Another study conducted in four hospitals in Kolkata, India, reported psychological harassment such as exhibitionism [43].

In our study, most harassers were Saudi patients and their victims were mostly non-Saudi healthcare workers. The systematic review on harassment against nurses also supports our results and mentions that patients were the most commonly reported harassers (review found 46% vs. our study found 33%), followed by physicians (41% vs. 28.5%), attendants (27.7% vs. 11.9%), and other health workers (17% vs. 18%) [5]. Similarly, a study done in another Saudi hospital found that most of the harassers were patients themselves [44]. There are different explanations for these findings. First, inappropriate actions (sexual harassment) from patients could come from their limited cognitive functions and lack of self-control [45]. It might also reflect the lack of attention toward the sexual needs of patients, such as adults with disabilities. Another important reason may be the language barrier between patients and healthcare providers which can lead to misunderstanding. Second, there are some weaknesses in the healthcare system due to which patients are unaware of the rights of healthcare workers. In addition, nurses work closely with patients and have more frequent contact with them compared to other healthcare workers [37]. Their job requires them to be more friendly and show sympathy toward patients, which places them in a vulnerable position and increases the risk to be harassed. Moreover, a study showed that a person with a friendly character had a nine-time higher risk of getting exposed to sexual harassment in comparison to an unfriendly attitude.

Concerning the place and time of reported harassment, our study found that the majority of incidents (54.5%) occurred in wards, which is consistent with another study conducted in Nepal [3]. This can be explained by the fact that in a majority of cases the patients were the harassers. Given the frequent and close contact with patients in the ward, it is the main location, as it was explained in previous studies that some patients who stay for a long time might take advantage of a therapeutic relationship [46]. Moreover, our study revealed that more than half of incidents (76%) happened during the day shift (between 8 am and 7 pm). This finding is in contradiction with a number of studies that found that most of the reported harassment acts occurred during the night shift (between 7 pm and 8 am) [19,20]. Reporting of harassment incidents during the daytime might be due to the presence of a large number of people and chaos during the day shift, or maybe the daytime data collection resulted in reporting of daytime incidents.

A Saudi study reported that sexual harassment and discrimination against residents resulted in a negative impact on their health and their ability to function [29]. The effect of sexual harassment on the victims was reported to cause a super alert and on-guard attitude after the experience [47]. A systematic review reported that 44.6% of the harassed nursing staff developed mental problems, 30.19% developed physical health problems, 61.26% developed emotional issues, 51.79% had psychological disturbance, and 16.02% had social health problems [38]. In our study, there was a significant association between women feeling depressed and hopeless with their experience of sexual harassment. Similar to our findings, an Egyptian study reported that 67.9% of the nurses who were sexually harassed suffered from depression [43]. A French study conducted among young physicians reported that there was an association between sexual harassment and increased anxiety and depression in the victims [48]. Another study conducted in Pakistan showed an association between depression and anxiety/stress with sexual harassment [49]. A meta-analysis of the negative consequences of the experience of sexual harassment showed that the victims had mental, physical, and psychological health issues, reflected as anxiety, depression, and even posttraumatic stress disorder [38]. Another study suggested that sexual harassment should be considered a traumatic event in an individual’s life [39].

The association between various low self-esteem items and sexual harassment was significant. It is clearly reflected in their negative feelings about having few good qualities, not being proud of themselves, having less respect for themselves, and increased feelings of failure. These findings are similar to a Pakistani study conducted on female trainee nurses in four hospitals that reported sexual harassment was significantly associated with low self-esteem and low job satisfaction [24]. Moreover, another study conducted among female family residents in the United States showed that the participants who reported sexual harassment suffered from low self-esteem, depression, and psychological impacts that required therapy [50]. A significant negative correlation was observed between K-10 scores and self-esteem scores (Pearson correlation r = -0.357, p < 0.00), suggesting a decrease in self-esteem with increasing depression. Hence, both distress and self-esteem may affect these women in a drastic way.

Most participants reported the incident to their friends or a family member outside the organization, while some reported to their colleagues within the organization, and only a few kept quiet. The differences in cultures play an important role in how the victims act or deal with the insults, and one of the positive things about Saudi culture is the strong connections and trust between family, friends, and colleagues which encourage people to talk and report such incidents. Similar results were reported in Malaysia and Nepal [12,13,40]. In contrast, some studies showed that the victims do not report such incidents to anyone [13,39], which may be further detrimental to their mental health.

This study has tried to comprehensively measure the association between mental health and sexual harassment; however, there are a few limitations. It is possible that response bias due to non-probability convenience sampling may have played a role as people who volunteered to participate may be different from those who opted not to participate (people might be scared to participate or report their experiences, afraid of being identified, etc.). Additionally, because the study period overlapped with the pandemic, it is possible that there was a shutdown of in-person services, resulting in under-reporting and under-participation. Similarly, the number of nurses who responded to the questionnaire was higher than the rest of the health workers which may have led to selection bias. Although the staff rotates during day/night shifts, the distribution of the questionnaire was mostly during the daytime shift, thus leaving out the night shift workers. Another limitation is that this study was conducted only at a tertiary care center; findings cannot be generalized to other centers/work environments and in rural areas. In addition to overcoming the above limitations, we suggest that male health workers are included as participants in future research studies.

## Conclusions

Addressing the incidents of sexual harassment in hospital settings should be prioritized for ensuring an effective workforce and high-quality health services. Harassment and mental well-being are closely associated. Understanding the negative impact of sexual harassment on the health of healthcare workers mandates effective preventive and workplace measures to be developed with a transparent system for reporting and addressing incidents of sexual harassment among all healthcare providers. Moreover, it is recommended that victims of sexual harassment should be counseled by professionals, such as psychologists or psychiatrists.
